# MiRNA Let-7a and Let-7d Are Induced by Globotriaosylceramide via NF-kB Activation in Fabry Disease

**DOI:** 10.3390/genes12081184

**Published:** 2021-07-30

**Authors:** Nadine Maier, Constantin Gatterer, Patrick Haider, Manuel Salzmann, Christoph Kaun, Walter S. Speidl, Gere Sunder-Plassmann, Bruno K. Podesser, Johann Wojta, Senta Graf, Max Lenz, Philipp J. Hohensinner

**Affiliations:** 1 Department of Internal Medicine II/Cardiology, Medical University of Vienna, 1090 Vienna, Austria; nadinemaier92@gmx.at (N.M.); constantin.gatterer@meduniwien.ac.at (C.G.); patrick.haider@meduniwien.ac.at (P.H.); manuel.salzmann@meduniwien.ac.at (M.S.); christoph.kaun@meduniwien.ac.at (C.K.); walter.speidl@meduniwien.ac.at (W.S.S.); johann.wojta@meduniwien.ac.at (J.W.); senta.graf@meduniwien.ac.at (S.G.); 2 Department of Internal Medicine III/Nephrology, Medical University of Vienna, 1090 Vienna, Austria; gere.sunder-plassmann@meduniwien.ac.at; 3 Center for Biomedical Research, Medical University of Vienna, 1090 Vienna, Austria; bruno.podesser@meduniwien.ac.at; 4 Ludwig Boltzmann Institute for Cardiovascular Research, 1090 Vienna, Austria

**Keywords:** Fabry disease, Gb3, NF-κB, inflammation

## Abstract

Background: Fabry disease is a hereditary genetic defect resulting in reduced activity of the enzyme α-galactosidase-A and the accumulation of globotriaosylceramide (Gb3) in body fluids and cells. Gb3 accumulation was especially reported for the vascular endothelium in several organs. Methods: Three Fabry disease patients were screened using a micro-RNA screen. An in vitro approach in human endothelial cells was used to determine miRNA regulation by Gb3. Results: In a micro-RNA screen of three Fabry patients undergoing enzyme replacement therapy, we found that miRNAs let-7a and let-7d were significantly increased after therapy. We demonstrate in vitro in endothelial cells that Gb3 induced activation of NF-κB and activated downstream targets. In addition, NF-κB activity directly reduced let-7a and let-7d miRNA expression as inhibiting NF-kB nuclear entry abolished the Gb3 effects. Conclusion: We suggest that let-7a and let-7d are potential markers for enzyme activity and inflammation in Fabry disease patients.

## 1. Introduction

Fabry disease is an X-linked inborn disease with a defective glycosphingolipid catabolism due to the deficient activity of α-galactosidase A [1]. This defect leads to an accumulation of respective substrates, mainly globotriaosylceramide (Gb3), in body fluids and cells [2]. Lyso-Gb3 can be considered as a reliable marker to confirm Fabry disease diagnosis [3]. Fabry disease is the second most common lysosomal storage disease with a worldwide incidence estimated to be around 1/40,000 to 1/117,000, not including late-onset disease [4]. The expected incidence of late-onset disease could be as high as 1/3100 [5]. Life expectancy of untreated patients was reported to be 58.2 years for males and 75.4 years for females with death mostly from cardiovascular disease [6]. The observed gender discrepancy is due to the lyonization process inactivating one X-chromosome leading to a usually milder disease progression including a delayed onset of disease in women [7].

A proinflammatory pattern with an increase in inflammatory cytokines was described for Fabry patients. Recent results suggested that the accumulation of Gb3 triggers a proinflammatory cascade mediated at least in part via toll-like receptor (TLR)4 [8]. TLR4 is a key receptor in pathogen recognition activated by damage-associated molecular patterns (DAMP). Downstream activation of NF-kB after TLR4 activation then leads to a pro-inflammatory response [9]. Therefore, the resulting pathogenesis of Fabry disease is partially dictated by the creation of a pro-inflammatory environment. Gb3 accumulation in patients was already associated with a prooxidative and apoptotic environment. Endothelial cells are especially a target of the prooxidative and proapoptotic environment generated by Gb3 [10]. In turn, due to proinflammatory activation of endothelial cells, adhesion proteins required for the interaction with mononuclear cells are upregulated, further fueling the inflammatory cascade. This proinflammatory state is directly reflected in the upregulation of proinflammatory cytokines and proteins but might also alter micro-ribonucleic acids (miRNAs) expression levels.

miRNAs are small, non-coding RNAs that bind to the 3′ untranslated region of mRNAs of specific genes to prevent their translation [11]. Therefore, miRNAs act as translational regulators. Given that miRNAs have a multitude of interaction partners, miRNAs can be viewed as regulatory nods responsible for a wider expression pattern regulation. Previous reports already suggested a different miRNA profile in patients with or without enzyme replacement therapy (ERT) [12].

The aim of our study was to identify miRNAs that are regulated by ERT and determine if these changes might be directly related to the disease.

## 2. Materials and Methods

### 2.1. miRNA Screening Assay

miRNA from three patients with Fabry disease before and after ERT and of three Fabry patients at two time points were isolated from EDTA plasma using an automated Maxwell system (Promega, Madison, WI, USA) with the respective miRNA isolation kit (miRNA tissue lysis kit, Promega) according to the manufacturer’s instructions. Blood samples were immediately processed and EDTA plasma fraction was stored at −80 °C as suggested by Glinge et al. [13]. miRNA panels were used and analyzed according to the suggestions from Qiagen (MIHS-3106ZG, Qiagen, Hilden, Germany,). Recruitment of patients followed the declaration of Helsinki including informed consent (Medical University of Vienna, Ethical Approval Number 813/2010).

### 2.2. Endothelial Cell Culture

Human umbilical vein endothelial cells (HUVEC) were isolated and propagated, as reported previously [14,15]. Endothelial cells were stimulated with 50 µM Gb3 (Matreya LLC, State College, PA, USA) and 50 µM dimethylfumerate (Sigma Aldrich, St. Louis, MO, USA) for the indicated time periods. A pool of 7 individual donors was used with all experiments performed at least three times (indicated in the figures).

### 2.3. qPCR

RNA extraction, as well as qPCR, were performed as published previously [16]. RNA from cell culture lysates was prepared using Maxwell RSC simplyRNA Tissue kits on a Maxwell RSC Instrument (both Promega, Madison, WI, USA). To create cDNA from the extracted RNA, 10 μL sample were added to 10 μL GoScript Reverse Transcriptase Mix (Promega, Madison, WI, USA) and reverse transcription was performed on a Thermocycler (Analytik Jena, Jena, Germany). Primers were designed using the Roche Universal ProbeLibrary (E-selectin forward primer 5′-accagcccaggttgaatg-3′, reverse primer 5′-ggttggacaaggctgtgc-3′, UPL Probe 86, amplicon size 89 bp; vascular cell adhesion protein 1 VCAM-1 forward primer 5′-tgaatctaggaaattggaaaaagg-3′, reverse primer 5′-tgaatctctggatccttaggaaa–3′, UPL probe 69, amplicon size 69 bp; intercellular adhesion molecule ICAM-1 forward primer 5′-ccttcctcaccgtgtactgg-3′, reverse primer 5′-agcgtagggtaaggttcttgc-3′, UPL Probe 71, amplicon size 90 bp; 18 s forward primer 5′-gcaattattccccatgaacg-3, reverse primer 5′-gggacttaatcaacgcaagc-3′, UPL Probe 48, amplicon size 82 bp). Amplification conditions consisted of an initial incubation at 95 °C for 10 min, followed by 45 cycles of 95 °C for 10 s, 63 °C for 20 s, and 72 °C for 6 s, followed by a final cooling phase of 40 °C. A BioRAD CFX Connect system with respective software was used (BioRAD, Hercules, CA, USA). Primers for let-7a and let-7d were purchased from Qiagen and qPCR was performed as indicated by the manufacturer.

### 2.4. Nuclear p50 ELISA

In order to determine the amount of the NF-kB component p50 translocation to the nucleus, we isolated nuclei from HUVEC using a nuclear extraction kit (Thermo Fisher, Waltham, MA, USA) according to the manufacturer’s instruction. P50 levels were determined using a commercially available ELISA with the DNA binding sequence of p50 as bait according to the manual (Abcam, Cambridge, UK).

### 2.5. Patients

The patients’ phenotypes were assigned to classical and non-classical FD according to the presence of specific signs and symptoms of the disease and the organ manifestations as suggested [17].

### 2.6. Statistics

Qiagen analysis software was used for miRNA screening analysis. For cell culture experiments a two-sided t-test was calculated with *p* < 0.05 considered significant using Graph Pad Prism (GraphPad, San Diego, CA, USA).

## 3. Results

To determine if Gb3 would have a direct effect on the composition of circulating miRNA in Fabry patients, we used a miRNA qPCR-based screening of 372 miRNAs in three previously treatment-naive patients before and during ERT. The mean time of ERT was 2.7 years before the second blood draw. Baseline characteristics of the patients are given in Table 1.

**Figure 1:**
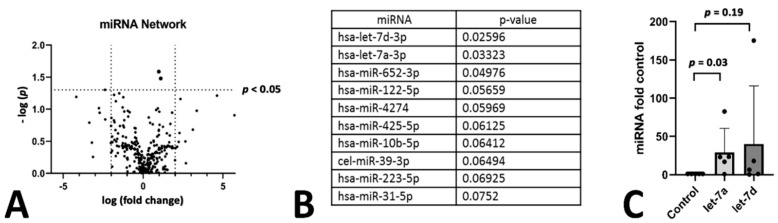
miRNA screening of Fabry patients. Screening of miRNA in Fabry Patients before and after enzyme replacement therapy. The difference in miRNAs in depicted in a vulcano plot (**A**) and the top 10 changed miRNAs are given in (**B**). The two top hits were analyzed in an age and sex matched cohort between untreated patients and patients under ERT therapy. Both values for let-7a and let-7d are given as fold control to the untreated matched control group (**C**).

We found that there were only few miRNAs affected by the treatment regime (Figure 1A,B). However, let-7a and let-7d belonging to the same family of miRNAs were significantly increased in patients during their ERT compared to before the start of the treatment. To determine if this change in the two miRNAs was dependent on the progression of the disease rather than the effect of therapy we also analyzed three patients at two different time points that did not receive therapy (let-7a was 6% reduced over baseline with *p* = 0.7 and let-7d was reduced by 23% over baseline with *p* = 0.99). In addition, we compared levels of miRNA let-7a and let-7d in five patients with ERT in comparison to five patients without (Figure 1C). In this small cohort, patients receiving ERT showed a significant increase of let-7a. Characteristics of this patient set is given in Table 2. In conclusion, let-7a and let-7d levels were unaffected in untreated patients over time.

**Figure 2:**
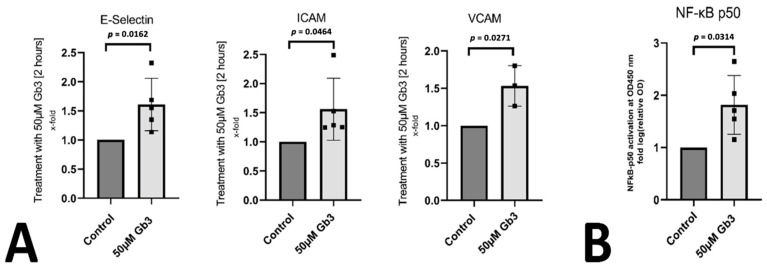
Gb3 induces a proinflammatory phenotype in endothelial cells: (**A**) human endothelial cells were stimulated with Gb3 and E-selectin, ICAM, and VCAM was determined by qPCR as indicated in the methods. Mean ± standard deviation is depicted with *p* ≤ 0.05 considered as significant. (**B**) p50 protein translocation after Gb3 stimulation was determined in nuclei of endothelial cells using a specific ELISA. Mean ± standard deviation is depicted with *p* ≤ 0.05 considered as significant.

To better understand the underlying direct pathogenic effects of accumulating Gb3 on inflammatory mechanisms, we used an in vitro cell culture approach using human umbilical vein endothelial cells (HUVEC) stimulated with Gb3 to simulate processes occurring in the vasculature in a Fabry patient. We were able to confirm a proinflammatory effect of Gb3 on endothelial cells, as the inflammation-related endothelial proteins E-selectin, ICAM, and VCAM were significantly upregulated at the mRNA level after 4 h of Gb3 treatment (Figure 2A). We confirmed this proinflammatory property of Gb3 by measuring the translocation of the NF-kB signaling component p50 into the nucleus after one hour of Gb3 treatment. Indeed, Gb3 significantly increased p50 protein in the nucleus of endothelial cells (Figure 2B).

**Figure 3:**
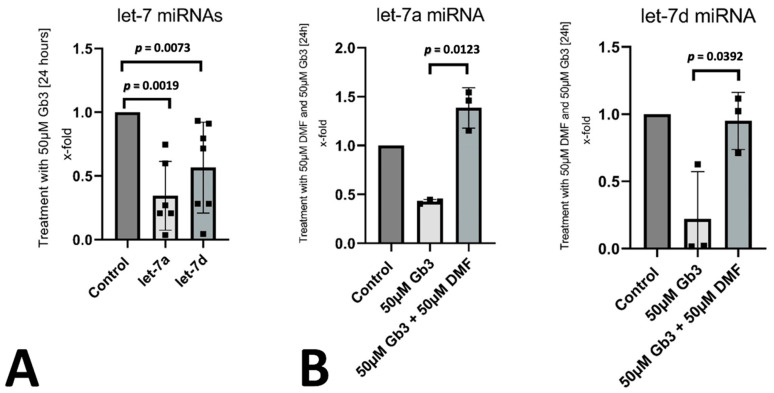
Regulation of let-7a and let-7d in vitro: (**A**) the influence of Gb3 on the expression of let-7a and let-7d was evaluated in endothelial cells. Mean ± standard deviation is depicted with *p* ≤ 0.05 considered as significant. (**B**) To determine the influence of NF-κB signaling on let-7a and let-7d expression levels, we used the NF-κB inhibitor dimethylfumerate (DMF) and evaluated the levels of let-7a and let-7d with and without inhibitor during Gb3 stimulation by qPCR. Mean ± standard deviation is depicted with *p* ≤ 0.05 considered as significant.

Let-7a and let-7d were previously described to be downregulated by NF-kB activation [13]. We found a significant downregulation of let-7a and let-7d after stimulation of endothelial cells with Gb3 (Figure 3A). To confirm the interplay between the two miRNAs with p50 activation, we used the NF-kB inhibitor dimethylfumerate to inhibit the translocation of NF-kB elements into the nucleus after Gb3 stimulation [18]. Inhibition of NF-kB abrogated the downregulation of let-7a and let-7d in endothelial cells suggesting a link between Gb3 activated NF-kB signaling and let-7a and let-7d reduction (Figure 3B).

## 4. Discussion

A miRNA screen in three human patients undergoing ERT for Fabry disease revealed an upregulation of let-7a and let-7d, which was previously demonstrated to be associated with inflammation [16]. In addition, we found in a small group of treated patients that let-7a levels were significantly increased over patients not receiving ERT. We confirmed a direct role of Gb3 in the reduction of let-7a and let-7d in endothelial cells in vitro and demonstrated the involvement of NF-κB signaling in the downregulation process. Our results confirm previous reports of a general proinflammatory response of cells to Gb3 stimulation [17].

Previous analysis of circulating miRNAs in Fabry disease patients mainly focused on comparing groups of patients with healthy control subjects or comparing treated and untreated patients [11,18]. The advantage of our analysis was the availability of samples of the same patient before and after ERT, therefore allowing us a more direct assessment of changes due to ERT. However, we only analyzed a limited number of patients, our results need to be validated in larger Fabry disease cohorts. Previously, mir-199a was associated with Fabry disease which was reported to be induced by inflammatory cytokines therefore supporting our observations of a proinflammatory milieu created by the enhanced circulation of Gb3 [18,19].

Interestingly, let-7a and let-7d were previously reported to be protective in a diabetic mouse model of advanced atherosclerosis by reducing proinflammatory cytokines [19]. In addition, let-7d was demonstrated to suppress inflammatory responses in neonatal rats with necrotizing enterocolitis [20]. In human patients, let-7d increase was a predictor for cirrhosis regression during treatment of a hepatits B virus infection [21]. Additional anti-inflammatory properties were also reported in microglia inflammation [22].

Overall, we suggest that let-7a and let-7d are directly regulated by Gb3 via the activation of the NF-kB pathway. As these two miRNAs can be measured in the circulation, we propose that analyzing the levels of these miRNAs might be beneficial in Fabry disease patients undergoing ERT as a possible measure for drug enzyme activity as targeting of the drug by the immune system might be reducing enzyme activity over time [20].

## Figures and Tables

**Table 1:** Baseline Fabry patient (screening group) characteristics.

Parameter	Patients under ERT (*n* = 3)	Patients without ERT (*n* = 3)
Gender	♀ (*n* = 2) ♂ (*n* = 1)	♀ (*n* = 2) ♂ (*n* = 1)
Age	40 ± 12 years	44 ± 17 years
Age at diagnosis	37 ± 11 years	41 ± 17 years
Follow-up time	3.3 years	4.3 years
eGFR (CKD-EPI)	55.42 mL/min/1.73 m^2^	93.68 mL/min/1.73 m^2^
Treatment	Agalsidase Alfa (0.2 mg/kg every other week) *n* = 2	-
	Agalsidase Beta (1 mg/kg every other week) *n* = 1	-
Variants and affected organs		
Patient #1	c.59C>A p.(Ala20Asp)	c.335G>A p.(Arg112His)
	Late-onset FD	Non-classic FD
	Heart, kidney	None
Patient #2	c.547+1G>C	c.335G>A p.(Arg112His)
	Classic FD	Non-classic FD
	Heart, kidney and nervous system	None
Patient #3	c.1132T>C p.(Cys378Arg)	c.335G>A p.(Arg112His)
	Late-onset FD	Non-classic FD
	Heart, kidney and nervous system	none

**Table 2:** Baseline Fabry patient (second group) characteristics.

Parameter	Patients under ERT (*n* = 5)	Patients without ERT (*n* = 5)
Gender	♀ (*n* = 2) ♂ (*n* = 3)	♀ (*n* = 4) ♂ (*n* = 1)
Age	39 ± 8.7 years	40 ± 9.2 years
Treatment	Agalsidase Alfa (0.2 mg/kg every other week)	-
Variants and affected organs	Reference sequence: NM_000169.2	
Patient #1	c.1288T>C p.(* 430GLnext)	c.195-2A>G
	Non-classical FD	Non-classical FD
	Kidney	Nervous system
Patient #2	c.319C>T p.(Gln107 *)	c.335G>A p.(Arg112His)
	Classical FD	Non-classical FD
	Heart kidney and nervous system	None
Patient #3	c.997C>T; p.(Gln333 *)	c.772G>A p.(Gly258Arg)
	Classical FD	Non-classical FD
	Heart, kidney and nervous system	None
Patient #4	c.758T>G p.(lle253Ser)	c.59>A p.(Ala20Asp)
	Classical FD	Non-classical FD
	Heart, kidney and nervous system	Heart and kidney
Patient #5	c.167_170delinsCCCT p.(Cys56_Gln57delinsSerLeu)	c.1132T>C p.(Cys378Arg)
	Classical FD	Non-classical FD
	Heart, kidney and nervous system	None

* refers to a stop codon.

## Data Availability

Research data is available from the corresponding authors upon reasonable request.
